# Symptoms of post-traumatic stress and associations with sexual behaviour and PrEP preferences among young people in South Africa, Uganda and Zimbabwe

**DOI:** 10.1186/s12879-022-07430-2

**Published:** 2022-05-16

**Authors:** Emily L. Webb, Janan J. Dietrich, Andrew S. Ssemata, Teacler G. Nematadzira, Stefanie Hornschuh, Ayoub Kakande, Gugulethu Tshabalala, Richard Muhumuza, Gertrude Mutonyi, Millicent Atujuna, Tarisai Bere, Linda-Gail Bekker, Melanie A. Abas, Helen A. Weiss, Janet Seeley, Lynda Stranix-Chibanda, Julie Fox, for the CHAPS Study Team

**Affiliations:** 1 MRC International Statistics and Epidemiology Group, London School of Hygiene and Tropical Medicine, WC1E 7HT London, UK; 2 Perinatal HIV Research Unit (PHRU), Faculty of Health Sciences, University of the Witwatersrand, Johannesburg, South Africa; 3 Health Systems Research Unit, South African Medical Research Council, Bellville, South Africa; 4 African Social Sciences Unit of Research and Evaluation (ASSURE), School of Clinical Medicine, Faculty of Health Sciences, University of the Witwatersrand, Johannesburg, South Africa; 5 MRC/UVRI and LSHTM Uganda Research Unit, Entebbe, Uganda; 6 Clinical Trials Research Centre, University of Zimbabwe, Harare, Zimbabwe; 7 Desmond Tutu HIV Centre, University of Cape Town, Cape Town, South Africa; 8 Institute of Psychiatry, Psychology and Neuroscience, King’s College London, London, UK; 9 Department of Global Health and Development, London School of Hygiene and Tropical Medicine, London, UK; 10 Child and Adolescent Health Unit, Faculty of Medicine and Health Sciences, University of Zimbabwe, Harare, Zimbabwe; 11 King’s College London, London, UK

**Keywords:** Post-traumatic stress disorder, Young people, Mental health, HIV, PrEP, Sub-Saharan Africa

## Abstract

**Background:**

It is not known whether post-traumatic stress disorder (PTSD) increases HIV-risk behaviours among young people in sub-Saharan Africa. We assessed associations of PTSD symptoms with sexual behaviour, HIV risk perception, and attitudes towards PrEP among young people taking part in the CHAPS community survey. We hypothesised that PTSD symptoms would increase sexual behaviours associated with HIV risk, hinder PrEP uptake and influence preference for daily versus on-demand PrEP.

**Methods:**

Young people without HIV, aged 13–24 years, were purposively recruited in Johannesburg and Cape Town in South Africa, Wakiso in Uganda, and Chitungwiza in Zimbabwe, and surveyed on socio-demographic characteristics, PrEP knowledge and attitudes, sexual behaviour, HIV perception and salience, and mental health. PTSD symptoms were measured using the Primary Care PTSD Screen for the Diagnostic and Statistical Manual of Mental Disorders 5 (PC-PTSD-5). Logistic and ordinal logistic regression was used to assess associations between PC-PTSD-5 score and socio-demographic characteristics, sexual behaviour, HIV risk perception, PrEP attitudes, and substance use, adjusting for age, sex, setting, depression and anxiety.

**Results:**

Of 1330 young people (51% male, median age 19 years), 522 (39%) reported at least one PTSD symptom. There was strong evidence that having a higher PC-PTSD-5 score was associated with reported forced sex (OR 3.18, 95%CI: 2.05–4.93), self-perception as a person who takes risks (OR 1.12, 95%CI: 1.04–1.20), and increased frequency of thinking about risk of HIV acquisition (OR 1.16, 95%CI: 1.08–1.25). PTSD symptoms were not associated with willingness to take PrEP, preference for on-demand versus daily PrEP, or actual HIV risk behaviour such as condomless sex.

**Conclusions:**

Symptoms consistent with probable PTSD were common among young people in South Africa, Uganda and Zimbabwe but did not impact PrEP attitudes or PrEP preferences. Evaluation for PTSD might form part of a general assessment in sexual and reproductive health services in these countries. More work is needed to understand the impact of PTSD on HIV-risk behaviour, forced sex and response to preventive strategies including PrEP.

**Supplementary information:**

The online version contains supplementary material available at 10.1186/s12879-022-07430-2.

## Background

HIV remains a major global public health problem; in 2019 an estimated 38 million people were living with HIV and there were an estimated 1.7 million new infections globally [1]. In sub-Saharan Africa (SSA) the number of people newly infected with HIV per year remains consistently high [2], with young people, particularly young women, experiencing the highest rates of infection [3]. This is despite the existence of effective HIV preventive strategies including pre-exposure prophylaxis (PrEP), which is recommended by the World Health Organisation (WHO) for persons at substantial risk of HIV acquisition as part of a combination preventive approach [4]. However, availability, user acceptability and uptake of PrEP is highly variable and often limited across the region, especially for young people [5].

As well as being a period of increased HIV acquisition risk, early adulthood is also the period in which mental disorders such as depression and anxiety frequently emerge [6]. Post-traumatic stress disorder (PTSD) [7], often as a consequence of sexual violence [8], is common in general population settings in SSA with a recent systematic review estimating a prevalence of probable PTSD of 22% [9]. Although comorbidity between PTSD and other mental disorders is common [10] and symptoms often overlap [11], PTSD occurring on its own may represent a different phenotype with distinct biological and psychological profiles compared to PTSD in the presence of co-morbid mental disorders [12].

Among young people in SSA, there is some, albeit inconclusive, evidence that common mental disorders such as depression and anxiety, and prior exposure to trauma including trauma due to sexual violence, may increase HIV-risk behaviours [13, 14] and HIV acquisition [15], and reduce HIV treatment adherence and outcomes [16, 17]. They may also impact adherence to PrEP for prevention of HIV, although evidence is mixed: a study in men who have sex with men and transgender women including participants from South Africa found that depression did not contribute to decreased adherence to daily PrEP on a population scale [18], but studies in Kenya and Uganda [19], and South Africa [20] found that depression was associated with poor adherence to daily PrEP among women but not among men. Limited research has been conducted among young people, although a study in the USA found that adolescents with and without mental disorders did not differ in PrEP retention outcomes [21]. Few studies have investigated whether PTSD, either in combination with other mental disorders or in isolation, impacts PrEP usage, and to our knowledge, no studies have examined the potential impact of PTSD on PrEP-related preferences in SSA.

The Combined HIV Adolescent PrEP and Prevention Study (CHAPS) is a mixed methods study conducted among young people in South Africa, Uganda and Zimbabwe [22]. It included a quantitative social science survey, with the overarching aim of identifying barriers and motivators towards the uptake of PrEP [22]. The survey, which included questions on PTSD symptoms, provided an opportunity to assess the primary objective of the current analysis: to assess whether probable PTSD was associated with attitudes towards PrEP, and with sexual behaviour and perceptions of HIV risk. Mental health disorders are known barriers to health care engagement and daily medication adherence, thus we hypothesise that probable PTSD may hinder willingness to take PrEP and disclose its usage, and influence preference for daily versus on demand PrEP. Evidence on this could have implications for how PrEP is offered to young people and whether a history of trauma would need to be taken into account when providing PrEP. We also hypothesise that probable PTSD may increase sexual risk behaviours associated with HIV risk, as has been previously demonstrated in other sub-Saharan African settings specifically for trauma due to sexual violence [13].

## Methods

### Study design

The primary aim of the current analysis was to assess associations of self-reported probable PTSD with sexual behaviour, perceptions of HIV risk, and attitudes towards PrEP among young people taking part in the CHAPS social science quantitative survey. Since PTSD occurring in the absence of other mental health co-morbidities may represent a different phenotype with distinct biological and psychological profiles compared to PTSD in the presence of co-morbid mental disorders, the secondary aim was to investigate whether other mental health co-morbidities modified any associations seen between probable PTSD and sexual behaviour, HIV risk and PrEP attitudes.

### Study setting and participants

Between March and December 2019, we used purposive community sampling [23] to recruit young people aged 13–24 years—prioritising those who were sexually active and living without known HIV—in four settings: Johannesburg and Cape Town in South Africa, Wakiso in Uganda, and Chitungwiza in Zimbabwe. Information on the study was first presented at community meetings organised with local officials and community mobilisers, who helped to identify locations at which young people congregated. CHAPS fieldworkers then visited these locations, coordinating with local leaders and community mobiliers (and Village Health Teams in Uganda) to provide information on the study, and to answer questions from potential participants and their parents/guardians. In South Africa, participants were recruited from community groups, schools, churches, bars, taxi ranks and other public meeting places. In Zimbabwe participants were recruited from community groups, youth centres, bars, voluntary counselling and testing sites, taxi ranks, and public meeting places. In Uganda, participants were recruited at fish landing sites.

Young people were eligible to participate in the survey if they were aged 13–24 years and willing to undergo HIV testing. In South Africa and Zimbabwe, this was administered as part of the screening and only young people without HIV were eligible for inclusion, while in Uganda, HIV status was not part of eligibility criteria, but recruited participants with reactive HIV test results (n = 9) based on samples obtained at enrolment, were not included in this analysis in order to ensure comparability in inclusion criteria across all settings.

### Ethical considerations

Ethical approval was obtained from the Human Research Ethics Committees of the University of Cape Town (290/2018) and the University of the Witwatersrand (180906B, M1811148 and 180,108) in South Africa, the Uganda Virus Research Institute Research and Ethics Committee (GC127/18/3/638) and Uganda National Council for Science and Technology (HS2534) in Uganda, the Joint Research Ethics Committee for the University of Zimbabwe, College of Health Sciences and the Parirenyatwa Group of Hospitals (JREC/195/18), the Medical Research Council of Zimbabwe and the Research Council of Zimbabwe (MRCZ/A/2356) in Zimbabwe, and the London School of Hygiene and Tropical Medicine Ethics Committee (15,629 and 16,182) in the UK.

Written, informed consent was obtained from all participants aged 18 years or over and written, informed parental consent and assent was obtained for participants aged under 18 years. A waiver of parental consent for emancipated minor participants was approved by ethical boards for use in Cape Town, Wakiso and Chitungwiza. Consent materials were available in Zulu and Sesotho in South Africa, Luganda in Uganda, Shona in Zimbabwe, and English in all settings. They were translated from English into other languages by bilingual outreach workers involved in HIV and adolescent programmes, followed by independent back translation, with further review and revision until agreement was reached, and final review by the above-named ethical review committees. Participants were reimbursed for participation-related time and travel costs following setting-specific national guidelines.

### Data collection and measures

Following written informed consent, and HIV testing, participants completed the quantitative survey. The survey was developed collaboratively with South African, Ugandan, Zimbabwean, and UK experts in adolescent health and HIV prevention and treatment, and was piloted among Adolescent Community Advisory Board members in each country. The survey was available in the same languages as described above for the consent materials, using the same approach to translation. Participants completed the survey on hand-held electronic devices in their language of choice at a private mutually convenient community location, with the support of trained interviewers available if necessary. The survey took approximately 45 min to complete and included questions on socio-demographic characteristics, knowledge and attitudes towards PrEP, past and current sexual behaviour characteristics, HIV perception and salience, mental health, and substance use, as described below.

Socio-demographic characteristics considered as exposures for this analysis were study setting, sex, age group, the highest level of education attended, whether the participant was the household head, age of household head, and the number of adults and rooms in the household. Data on historical sexual behaviour included age of first sex and any history of transactional sex. Information collected on recent or current sexual behaviour included forced sex or forcing sex in the last 6 months, number of partners in the last 6 months, sex frequency in the past month, degree of advance knowledge of the most recent sexual encounter, and current relationship status. Information on age, relationship, type, condom use, and HIV status of most recent partner was also collected. Participants were asked about whether they perceived themselves generally as a person who takes risks through two questions: (1) “Are you generally a person who takes risks? I am going to read three answer choices and I would like you to tell me which one is closest to the truth about you personally”﻿, with possible responses “I take risks”, “I am somewhere in between”, “I avoid taking risks”, and (2) “Please rate yourself from 0 to 10, where 0 means you are unwilling to take any risks and 10 means you are always willing take risks”. They were also asked about how often they had thought about the risk of acquiring HIV in the last 3 months, and how likely they felt they were to become infected with HIV in the next 3 months. Regarding PrEP, the two main ways in which PrEP can be taken were described to the participants as i) Daily PrEP: one pill every day whether you are having sex or not; and ii) On-demand PrEP: taking two pills before you have sex and two pills after sex. Participants were asked whether they felt they would prefer on-demand or daily PrEP, whether they would choose to take PrEP if it cost the same amount as a hot meal and whether they would disclose PrEP use to their partner.

Information on exposure to traumatic events and post-traumatic stress symptoms was collected using the Primary Care PTSD Screen for *DSM-5* (PC-PTSD-5), a 5-item screen related to the Diagnostic and Statistical Manual of Mental Disorders (DSM-5) diagnosis criteria for PTSD that was designed for use in primary care settings to identify respondents with probable PTSD [24]. Respondents indicating no exposure to any traumatic event throughout their life are assigned a score of 0. Respondents reporting lifetime exposure to any traumatic event are asked five additional binary questions about how that trauma exposure has affected them over the past month. The number of positive responses are added to give a total PC-PTSD-5 score. Since the tool has not been validated in SSA youth (and an appropriate cut-off has not been determined), the total PC-PTSD-5 score was used for primary analysis. In secondary analysis, we also investigated using a PC-PTSD-5 cut-off score of ≥ 3 which has been shown to have 90% sensitivity and 80% specificity as a screen for PTSD in US army veterans [25], to perform well for detecting probable PTSD among young people in the US [26] and has been previously implemented, although not validated, among young people in South Africa [27]. Following completion of PC-PTSD-5 questions, study staff did not have access to participants’ responses but asked participants if there were any experiences or feelings that they would like to discuss; those who said they did want to talk to someone could receive counselling from trained staff at the study clinic or clinic close to the location of interview.

As previously described [28], information on depressive symptoms was collected using the 9-item Patient Health Questionnaire (PHQ-9) [29]. Participants were classified as having moderate/severe depressive symptoms if their PHQ-9 score was ≥ 10. The PHQ-9 is validated in many languages including those used in the CHAPS study [30–32]. Generalised anxiety symptoms were assessed using the Generalized Anxiety Disorder-2 item tool (GAD-2) [33], with participants with a score ≥ 3 classified as having anxiety symptoms. The GAD-2 tool is validated in many languages including those used in the CHAPS study [34, 35]. Information on alcohol use was collected as the frequency of binge drinking, defined as 6 or more drinks. Information on whether other substances had been used in the last 30 days was also collected.

### Statistical analysis

Statistical analysis was done using Stata version 16. The sample size provides 90% power to detect a difference in proportion with PC-PTSD-5 ≥ 3 of 22% vs. 15% for exposures with 50% prevalence. Responses for the PC-PTSD-5 tool were summarised for all participants and separately by study setting. Since data were collected using a cross-sectional study design, it is not possible to determine causality. For associations between PTSD score and socio-demographic characteristics, PC-PTSD-5 score was considered as the outcome of interest, and related to socio-demographic characteristics using ordinal logistic regression to obtain crude and adjusted odds ratios (OR) and 95% confidence intervals (CI), and the likelihood ratio test to obtain p-values. For examining associations of PTSD score with sexual behaviour, HIV risk perception, attitudes towards PrEP, and substance use, PC-PTSD-5 score was included as the exposure of interest in separate regression models for each of these outcomes. Logistic regression and ordinal logistic regression models were fitted for binary and ordered categorical outcomes, respectively. The proportional odds assumption for ordinal logistic regression models was tested. For self-perception as a person who takes risks score measured on a continuous scale from 0 to 10, linear regression was used to assess the association with PC-PTSD-5 score. In secondary analyses, PC-PTSD-5 score was classified as ≥ 3 versus < 3 and modelled as a binary outcome variable (for associations with socio-demographica characteristics) or a binary exposure variable (for associations with sexual behaviour, HIV risk perception, attitudes towards PrEP and substance use).

For all analyses, multivariable regression models controlled for age, sex and study setting. For analyses including PC-PTSD-5 score as the exposure of interest, and measures of sexual behaviour, HIV risk perception and attitudes towards PrEP as outcomes of interest, regression models further adjusted for depression and anxiety to assess whether these acted as possible confounders of any associations. Finally, the role of these other mental health co-morbidities as potential modifiers of the relationship of PC-PTSD-5 score with sexual behaviour, HIV risk perception and PrEP attitudes was assessed, by fitting interaction terms between PC-PTSD-5 score and a binary variable for depression and/or anxiety to assess whether PC-PTSD-5 score and depression/anxiety combined multiplicatively in their impact on outcomes. A likelihood ratio test was used to formally test for interaction with a p-value of less than 0.05 interpreted as evidence for effect modification.

## Results

A total of 1330 participants aged 13–24 years (mean: 19 years) took part in the survey. Table 1 summarises responses to the PC-PTSD-5 tool, overall and by study setting. A total of 653 participants (49%) reported experiencing a traumatic event during their lifetime. Of these, the majority (522, 80%) reported at least one symptom of post-traumatic stress that they had experienced in the last month and that they associated with that event. A total of 254 (19%) had PC-PTSD-5 ≥ 3.

**Table 1:** Responses to Primary Care PTSD Screen for *DSM-5* (PC-PTSD-5) questions, overall and by study setting

Question	Cape Town (n = 239)	Johannesburg (n = 200)	Entebbe (n = 491)	Chitungwiza (n = 400)	Total (n = 1330)
Have you ever experienced a traumatic event?					
No	95 (40%)	95 (48%)	243 (49%)	244 (61%)	677 (51%)
Yes	144 (60%)	105 (53%)	248 (51%)	156 (39%)	653 (49%)
In the past month, have you experienced nightmares about the event?^a^					
No	68 (47%)	60 (57%)	144 (58%)	79 (51%)	351 (54%)
Yes	76 (53%)	45 (43%)	105 (42%)	77 (49%)	302 (46%)
In the past month, have you tried hard not to think about the event or avoided situations that remind you of the event?^a^					
No	41 (28%)	40 (38%)	126 (51%)	54 (35%)	261 (40%)
Yes	103 (72%)	65 (62%)	122 (49%)	102 (65%)	392 (60%)
In the past month, have you been constant on guard, watchful or easily startled?^a^					
No	94 (65%)	63 (60%)	159 (64%)	107 (69%)	423 (65%)
Yes	50 (35%)	42 (40%)	89 (36%)	49 (31%)	230 (35%)
In the past month, have you felt numb or detached from people, activities or your surroundings?^a^					
No	79 (55%)	57 (54%)	192 (77%)	96 (62%)	424 (65%)
Yes	65 (45%)	48 (46%)	56 (23%)	60 (38%)	229 (35%)
In the past month, have you felt guilty or unable to stop blaming yourself or others for the event?^a^					
No	88 (61%)	63 (60%)	184 (74%)	99 (63%)	434 (66%)
Yes	56 (39%)	42 (40%)	64 (26%)	57 (37%)	219 (34%)
Total scale score					
0	107 (45%)	111 (56%)	324 (66%)	266 (67%)	808 (61%)
1	25 (10%)	13 (7%)	46 (9%)	24 (6%)	108 (8%)
2	42 (18%)	29 (15%)	42 (9%)	47 (12%)	160 (12%)
3	30 (13%)	22 (11%)	28 (6%)	32 (8%)	112 (8%)
4	24 (10%)	20 (10%)	34 (7%)	24 (6%)	102 (8%)
5	11 (5%)	5 (3%)	17 (3%)	7 (2%)	40 (3%)
Post-traumatic stress symptoms score, categorised					
PC-PTSD-5 score < 3	174 (73%)	153 (77%)	412 (84%)	337 (84%)	1076 (81%)
PC-PTSD-5 score ≥ 3	65 (27%)	47 (24%)	79 (16%)	63 (16%)	254 (19%)

^a^Among those who reported ever experiencing a traumatic event

Table 2 shows associations between socio-demographic characteristics and historical sexual behaviour, and PC-PTSD-5 score. PC-PTSD-5 scores were higher on average among participants in both South African settings (particularly Cape Town) and lower among participants in Wakiso and Chitungwiza. There was no evidence of association of PC-PTSD-5 score with sex, age group, education, number of rooms in the household, or number of adults per room in the household. However, there was some evidence that participants who were household heads, who had a higher number of adults in their households, or who had older household heads had higher PC-PTSD-5 scores (Table 2). After adjusting for age, sex and study setting, there was no evidence of an association with PC-PTSD-5 score for ever having sex or age at first sex. Participants reporting ever having had transactional sex were more likely to have a higher PC-PTSD-5 score (adjusted OR 1.89, 95% CI 1.34–2.66, p < 0.001). There was strong evidence that forced sex was associated with PC-PTSD-5 score in both crude and adjusted analyses (adjusted ORs 3.18, 95% CI: 2.05–4.93 and 2.93, 95% CI: 1.74–4.94 for being forced to have sex and forcing someone else to have sex, respectively; p < 0.001).

**Table 2:** Associations between socio-demographic characteristics, historical sexual behaviour and post-traumatic stress symptoms

Characteristic	Category	PC-PTSD score						Crude results		Adjusted results^a^	
		0	1	2	3	4	5	OR (95% CI)	p	OR (95% CI)	p
Study setting	Cape Town	107 (45%)	25 (10%)	42 (18%)	30 (13%)	24 (10%)	11 (5%)	2.21 (1.65, 2.97)	< 0.001	2.25 (1.67, 3.02)	< 0.001
	Johannesburg	111 (56%)	13 (7%)	29 (15%)	22 (11%)	20 (10%)	5 (3%)	1.58 (1.15, 2.19)		1.59 (1.15, 2.19)	
	Entebbe	324 (66%)	46 (9%)	42 (9%)	28 (6%)	34 (7%)	17 (3%)	Baseline		Baseline	
	Chitungwiza	266 (67%)	24 (6%)	47 (12%)	32 (8%)	24 (6%)	7 (2%)	0.98 (0.75, 1.29)		0.99 (0.75, 1.30)	
Sex	Male	405 (60%)	69 (10%)	96 (14%)	47 (7%)	42 (6%)	14 (2%)	Baseline	0.39	Baseline	0.37
	Female	403 (61%)	39 (6%)	64 (10%)	65 (10%)	60 (9%)	26 (4%)	1.10 (0.89, 1.36)		1.10 (0.89, 1.37)	
Age group	13–15	90 (60%)	15 (10%)	25 (17%)	11 (7%)	8 (5%)	1 (1%)	0.89 (0.64, 1.25)	0.43	0.83 (0.60, 1.17)	0.29
	16–17	152 (63%)	24 (10%)	25 (10%)	12 (5%)	23 (10%)	4 (2%)	0.84 (0.63, 1.11)		0.82 (0.62, 1.10)	
	18–24	566 (60%)	69 (7%)	110 (12%)	89 (9%)	71 (8%)	35 (4%)	Baseline		Baseline	
Highest level of education attended	Still studying	420 (60%)	65 (9%)	89 (13%)	53 (8%)	52 (7%)	17 (2%)	Baseline	0.45	Baseline	0.96
	≤ Grade 7	85 (66%)	9 (7%)	15 (12%)	7 (5%)	8 (6%)	4 (3%)	0.80 (0.54, 1.18)		0.97 (0.63, 1.48)	
	Grade 7–12	274 (60%)	31 (7%)	53 (12%)	44 (10%)	42 (9%)	15 (3%)	1.10 (0.88, 1.39)		1.06 (0.82, 1.38)	
	Post-school	29 (62%)	3 (6%)	3 (6%)	8 (17%)	0 (0%)	4 (9%)	1.06 (0.59, 1.92)		1.00 (0.55, 1.84)	
Participant is household head	No	714 (61%)	89 (8%)	143 (12%)	99 (8%)	89 (8%)	32 (3%)	Baseline	0.38	Baseline	0.03
	Yes	94 (57%)	19 (12%)	17 (10%)	13 (8%)	13 (8%)	8 (5%)	1.15 (0.84, 1.58)		1.47 (1.04, 2.08)	
Household head age	Per unit increase							1.02 (1.01, 1.02)	0.001	1.01 (1.00, 1.02)	0.02
Number of adults in household^b^	1–2	266 (67%)	29 (7%)	38 (10%)	29 (7%)	22 (6%)	13 (3%)	Baseline	0.002	Baseline	0.05
	3–4	376 (60%)	42 (7%)	85 (14%)	52 (8%)	53 (9%)	14 (2%)	1.32 (1.02, 1.71)	< 0.001^f^	1.26 (0.95, 1.66)	0.01^f^
	5+	165 (53%)	37 (12%)	37 (12%)	31 (10%)	27 (9%)	13 (4%)	1.69 (1.26, 2.26)		1.49 (1.09, 2.03)	
Number of rooms in household	1–2	298 (65%)	33 (7%)	50 (11%)	35 (8%)	26 (6%)	13 (3%)	Baseline	0.03	Baseline	0.24
	3–4	240 (57%)	41 (10%)	57 (14%)	32 (8%)	38 (9%)	13 (3%)	1.39 (1.07, 1.80)		1.26 (0.95, 1.66)	
	5+	270 (59%)	34 (7%)	53 (12%)	45 (10%)	38 (8%)	14 (3%)	1.31 (1.01, 1.71)		1.20 (0.90, 1.60)	
Number of adults per room in household^b^	< 1	325 (60%)	44 (8%)	66 (12%)	49 (9%)	40 (7%)	15 (3%)	Baseline	0.66	Baseline	0.89
	≥ 1 and < 2	320 (60%)	40 (8%)	66 (12%)	42 (8%)	44 (8%)	19 (4%)	1.02 (0.81, 1.30)		1.02 (0.80, 1.31)	
	≥ 2	162 (63%)	24 (9%)	28 (11%)	21 (8%)	18 (7%)	6 (2%)	0.90 (0.67, 1.20)		0.95 (0.69, 1.30)	
Ever had sex^c^	No	196 (70%)	19 (7%)	29 (10%)	16 (6%)	15 (5%)	5 (2%)	Baseline	< 0.001	Baseline	0.24
	Yes	611 (58%)	89 (9%)	131 (13%)	95 (9%)	86 (8%)	35 (3%)	1.67 (1.27, 2.21)		1.22 (0.87, 1.71)	
Age of first sex	Per unit increase							0.95 (0.91, 0.99)	0.02	0.96 (0.91, 1.01)	0.12
Transactional sex, ever^d^	No	748 (63%)	92 (8%)	139 (12%)	92 (8%)	91 (8%)	33 (3%)	Baseline	< 0.001	Baseline	< 0.001
	Yes	57 (45%)	16 (13%)	19 (15%)	19 (15%)	9 (7%)	7 (6%)	1.84 (1.32, 2.57)		1.89 (1.34, 2.66)	
Forced sex, last 6 months^e^	No	777 (62%)	102 (8%)	152 (12%)	101 (8%)	84 (7%)	34 (3%)	Baseline	< 0.001	Baseline	< 0.001
	Yes	30 (39%)	6 (8%)	7 (9%)	10 (13%)	17 (22%)	6 (8%)	3.06 (1.98, 4.71)		3.18 (2.05, 4.93)	
Forcing sex, last 6 months^e^	No	788 (62%)	102 (8%)	154 (12%)	105 (8%)	91 (7%)	35 (3%)	Baseline	< 0.001	Baseline	< 0.001
	Yes	19 (37%)	6 (12%)	5 (10%)	6 (12%)	10 (20%)	5 (10%)	2.99 (1.78, 5.01)		2.93 (1.74, 4.94)	

^a^Adjusted for study setting, sex and age group

^b^1 missing value

^c^3 preferred not to say

^d^8 preferred not to say

^e^4 preferred not to say

^f^Test for trend

Table 3 shows associations between PC-PTSD-5 score as the exposure variable of interest with outcomes of sexual behaviour characteristics, perceptions of HIV risk, attitudes towards PrEP, and drug and alcohol use. After adjusting for study setting, sex and age, group, a higher PC-PTSD-5 score was associated with increased number of partners, and increased odds of the last sexual encounter being with a non-regular partner. There was no evidence of association with any other sexual behaviour characteristics (Table 3).

**Table 3:** Associations between post-traumatic stress symptom score (PC-PTSD-5 ≥ 3 versus PC-PTSD-5 < 3) and sexual behaviour, PrEP attitudes and mental health co-morbidities

Outcome variable and categories^a^	Crude results		Adjusted for study setting, sex and age group		Further adjusted for depression and anxiety	
	OR^b^ (95% CI)	p-value	OR^b^ (95% CI)	p-value	OR^b^ (95% CI)	p-value
Sexual behaviour characteristics						
Number partners, last 6 months (0, 1, 2, 3+)	1.15 (1.08, 1.23)	< 0.001	1.08 (1.01, 1.16)	0.02	1.08 (1.01, 1.16)	0.03
Sex frequency, past month (never, weekly-monthly, >weekly)	1.14 (1.06, 1.22)	< 0.001	1.04 (0.96, 1.12)	0.32	1.03 (0.96, 1.11)	0.41
Last time had sex, how far in advance knew (> 24 h, 12–23 h, 2–12 h, < 2 h)	1.04 (0.97, 1.12)	0.27	1.02 (0.95, 1.10)	0.55	1.02 (0.95, 1.10)	0.55
Condom use last sex with recent partner (no, yes)	0.94 (0.87, 1.02)	0.14	0.93 (0.85, 1.01)	0.09	0.95 (0.87, 1.03)	0.23
Type of relationship, most recent partner (regular, casual/paying/other)	1.11 (1.00, 1.22)	0.04	1.15 (1.03, 1.28)	0.01	1.14 (1.02, 1.27)	0.02
HIV status, most recent partner (negative, positive/don’t know)	1.07 (0.98, 1.15)	0.12	1.09 (1.00, 1.18)	0.05	1.09 (1.00, 1.19)	0.05
General risk taking and HIV salience characteristics						
Self-perception of frequency of risk-taking (never, sometimes, often)	1.16 (1.09, 1.25)	< 0.001	1.13 (1.05, 1.21)	0.001	1.12 (1.04, 1.20)	0.004
Thought about risk of HIV, last 3 months (never, rarely, sometimes, often)	1.23 (1.15, 1.31)	< 0.001	1.18 (1.10, 1.27)	< 0.001	1.16 (1.08, 1.25)	< 0.001
Chance of HIV, next 3 months (none, some, moderate/high)	1.16 (1.08, 1.24)	< 0.001	1.10 (1.03, 1.19)	0.01	1.09 (1.01, 1.17)	0.02
Attitudes towards PrEP						
PrEP preference (on demand, daily)	1.09 (1.01, 1.18)	0.02	1.04 (0.96, 1.13)	0.32	1.04 (0.96, 1.13)	0.36
Would take PrEP if same price as hot meal (no, yes)	1.08 (0.99, 1.17)	0.07	1.06 (0.97, 1.15)	0.18	1.07 (0.99, 1.17)	0.10
Would disclose PrEP to partner (no, yes)	1.03 (0.95, 1.12)	0.42	1.01 (0.93, 1.09)	0.85	1.02 (0.94, 1.11)	0.58
Alcohol and drug use						
Frequency of binge drinking (don’t drink; never, <monthly, ≥monthly)	1.21 (1.14, 1.30)	< 0.001	1.14 (1.06, 1.23)	< 0.001	1.14 (1.06, 1.23)	0.001
Drug use, last 30 days (no, yes)	1.14 (1.03, 1.26)	0.01	1.08 (0.96, 1.22)	0.19	1.06 (0.94, 1.19)	0.34

^a^For binary outcome variables, the reference group is listed first; for ordered categorical outcome variables, the categories are listed in order;

^b^Odds ratio represent the increase in odds for a one unit increase in PC-PTSD-5 score

There was strong evidence that PC-PTSD-5 score was associated with increased self-perceived risk taking, both assessed on a three-point Likert scale (OR 1.12, 95% CI: 1.04–1.20, p = 0.004) and as a continuous scale between 0 (unwilling to take risks) and 10 (always willing to take risks): adjusted difference in mean self-perceived risk-taking score 0.16 (95% CI: 0.07–0.26, p = 0.001) for each unit increase in PC-PTSD-5 score. There was also strong evidence that PC-PTSD-5 score was associated with the frequency of thinking about the risk of HIV in the last 3 months (OR 1.16, 95% CI: 1.08–1.25, p < 0.001) and with perceived chance of acquiring HIV in the next 3 months (OR 1.09, 95% CI: 1.01–1.17, p = 0.02). In adjusted analyses, there was no evidence of an association of PC-PTSD-5 score with preference for daily versus on demand PrEP, willingness to take PrEP if it cost the same as a hot meal, willingness to disclose PrEP use to a partner, or drug use but there was evidence of an association with increased frequency of binge drinking, both before and after adjusting for depression and anxiety (adjusted OR 1.14, 95% CI: 1.06–1.23, p = 0.001). Results from analyses analysing PC-PTSD-5 score as a binary variable (≥ 3 versus < 3) were consistent (Additional file 1: Tables S1, S2).

**Fig. 1:**
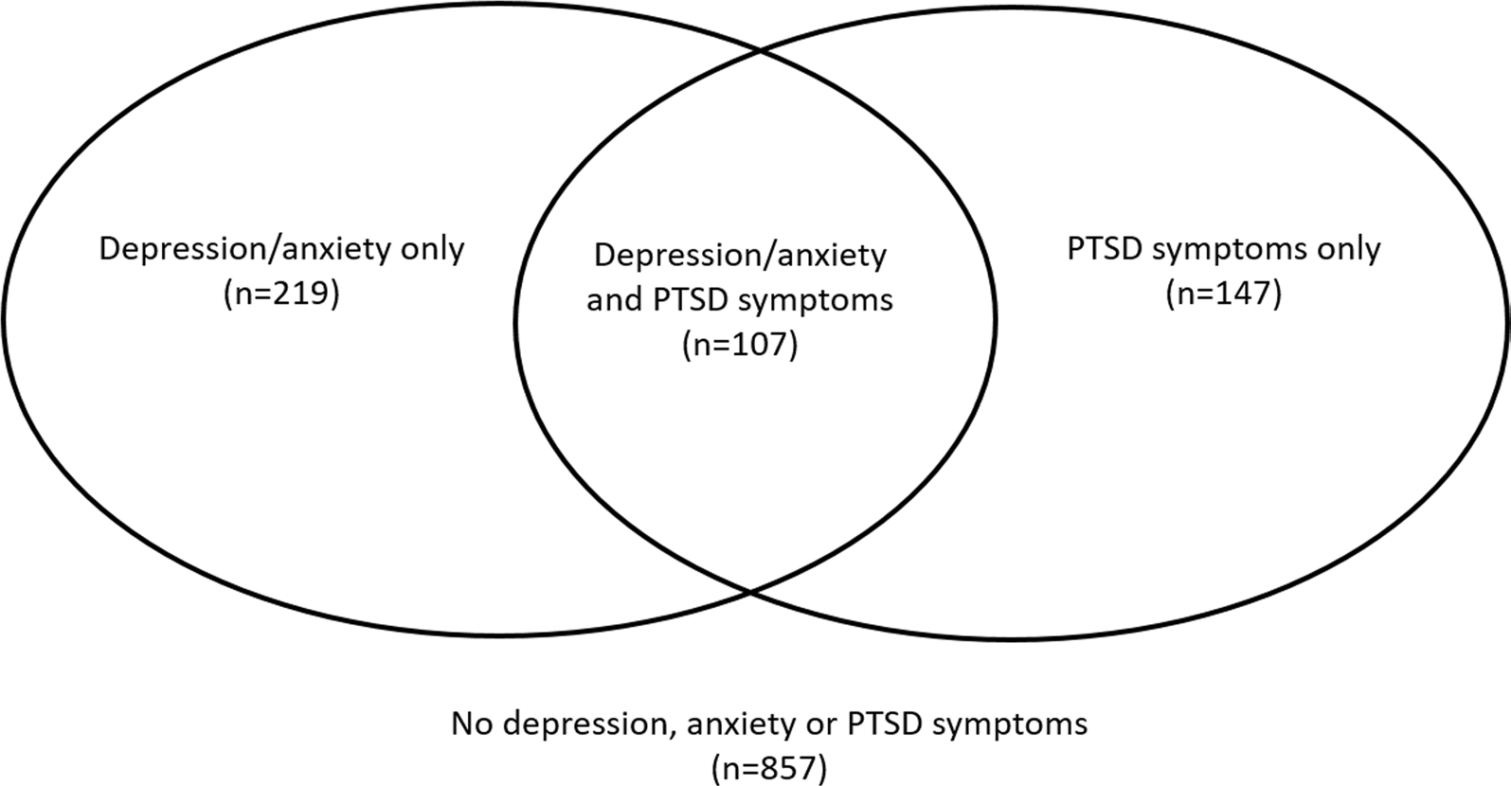
The overlap between PTSD, depression and anxiety in CHAPS survey participants

Of the 254 participants with PC-PTSD-5 ≥ 3, 147 (57%) did not report symptoms of either depression or anxiety (Fig. 1). As noted above, adjustment for these co-morbidities had little impact on most associations reported. We included interaction terms to assess whether the association of PC-PTSD-5 score with outcomes differed in the presence (versus absence) of these other mental health co-morbidities. For nearly all outcomes assessed, there was no evidence that their association with PC-PTSD-5 score differed in the presence versus absence of depression and/or anxiety. The only exception was for the type of relationship with the most recent partner (interaction p-value 0.02): among people who had depression or anxiety, PC-PTSD-5 score was associated with higher odds of casual sex (adjusted OR 1.35, 95% CI: 1.13–1.61) but there was no association of PC-PTSD-5 score with causal sex among those who did not have depression or anxiety (adjusted OR 1.03, 95% CI: 0.89–1.19).

## Discussion

In this large cross-sectional study of young people aged 13–24 years from four different settings in South Africa, Uganda and Zimbabwe, symptoms of post-traumatic stress disorder were common. Young people with a higher PC-PTSD-5 score had increased odds of reporting forced sex or forcing someone else to have sex. PTSD symptoms were also associated with increased number of sexual partners, recent sex with a non-regular partner, increased self-perception as a person who takes risks, with the frequency of thinking about the risk of HIV, and with binge drinking. However, contrary to our hypotheses, after controlling for confounders we did not find evidence that PTSD symptoms were associated with willingness to take PrEP, disclose PrEP use or preference for daily versus on-demand PrEP.

The level of PTSD symptoms reported in CHAPS survey participants is consistent with estimates from other general population settings in SSA [9], and from youth in low- and middle-income countries [36]. Our results also align with findings from South Africa suggesting that links between probable PTSD and other socio-demographic factors such as education are not as evident as in high-income countries [37]. The high level of PTSD symptoms in this sample of young people, with the caveat that this is a non-probability based sample and thus study participants are not representative of all youth in these settings, suggests that evaluation for PTSD should form part of a general assessment in sexual and reproductive health services in these countries.

PTSD symptoms often co-existed with depression and anxiety in our study participants, however around 60% of participants with a PC-PTSD-5 score ≥ 3 were not classified as having either depression or anxiety, a higher proportion than was observed in a study among youth from a European setting [38], which assessed mental disorders using a diagnostic interview. PTSD symptoms remained independently and strongly associated with forced sex, self-perception as a person who takes risks, and frequency of thinking about the risk of HIV, regardless of whether or not the participant also had symptoms of depression or anxiety. In other words, even among participants who did not have depression or anxiety, PTSD symptoms were associated with these outcomes. However, there was a suggestion of an association between PTSD symptoms and casual sex only among those with depression or anxiety, which was not seen among those without these co-morbidities.

Contrary to our hypothesis, we found little evidence that PTSD symptoms were associated with willingness to take PrEP, willingness to disclose PrEP usage or preference for on-demand versus daily PrEP. Furthermore, results from the same study demonstrated that depression and anxiety symptoms (without consideration of PTSD score) were similarly not associated with willingness to take PrEP or with preference for on demand versus daily PrEP, although they were associated with participant-held concerns that they would be more likely to have riskier sex if they were to take PrEP [28]. Possible explanations for this lack of association include the fact that many participants were unfamiliar with PrEP before taking part in the survey and this may have precluded them from forming opinions on PrEP that could in turn be influenced by any underlying mental health conditions. Furthermore, since both daily and on demand PrEP regimens were described to participants before collecting information on their attitudes towards PrEP, it is plausible that PrEP might not be considered in the same way as medications which are designed to be taken daily, such as ARVs for which previous evidence has suggested that trauma may be a barrier to adherence. However, given the limitations discussed below with ascertaining PTSD, further work is needed to understand whether past trauma should be specifically considered when delivering PrEP.

We found some evidence that PTSD symptoms were associated with sexual behaviour, consistent with previous findings which have related increased traumatic experience exposure to high risk sexual behaviour. For example, a positive association between childhood sexual violence and infrequent condom use and an increased number of partners has been reported among young people in Tanzania [13]. In South Africa lifetime traumatic experiences have been correlated with transactional sex and the number of sexual partners [39]. Since this was a cross-sectional survey, we were not able to assess causality, however the observed association of probable PTSD with forced sex may indicate that probable PTSD occurred as a consequence of forced sex for some participants.

A key limitation of this work is that it did not use a tool that has been validated for assessing probable PTSD in this population. As described previously [22], the overarching aim of the CHAPS survey was to identify barriers and motivators towards the uptake of PrEP, of which PTSD was one of several considered; thus the survey tool included only a brief screen for traumatic experiences and consequent feelings, which has been validated for use in identifying US veterans with probable PTSD [24], and validated among young people in the US [26] and previously used but not validated in young people in SSA [27]. For this reason, our primary analysis is based on the PC-PTSD-5 score rather than based on a specific cutpoint for this tool. We also did not collect information on the types of traumatic experiences that led to the reporting of symptoms. Other studies have shown that childhood traumas are highly prevalent among young people in urban communities in South Africa [14] and associated with PTSD [40]; exposure to violence, particularly sexual violence is also common [41, 42]. However, in the absence of a more in-depth assessment, we cannot speculate on the possible causes of probable PTSD.

Further limitations of the analysis include the cross-sectional study design, which precludes conclusions about the direction of causation. Participants were recruited into the study through a community outreach approach, and not through a probability-based sampling approach. Thus they are not be representative of all young people in their communities, so that our results may not be generalisable to all young people in these settings, and we cannot make comparisons between the different study settings in terms of prevalence. This also means that we cannot obtain an estimate of response rate, thus we cannot assess whether characterstics of our participants were similar to those of others who were made aware of the study but chose not to take part. Data collected were self-reported, which could have introduced measurement bias, with some behavioural variables in particularly likely to be subject to underreporting. Our study had several strengths including the large sample size which provided good power to detect associations, and the inclusion of participants from different settings in SSA.

## Conclusions

In summary, PTSD symptoms were common in young people in South Africa, Uganda and Zimbabwe and associated with forced sex but not with other sexual behaviour characteristics or with attitudes towards PrEP. Further work should employ a PTSD tool that has been specifically validated in the target population, and should also assess possible causes of PTSD. Although mental health screening for depression and anxiety should be incorporated into PrEP counselling as it can impact adherence to PrEP [19, 20], the impact of PTSD on PrEP adherence is not known and needs investigation. The large proportion of young people with symptoms suggestive of probable PTSD in the absence of anxiety or depression suggests that young people should be screened for PTSD as well as for other common mental disorders. Such evaluation might form part of a general assessment in sexual and reproductive health services in these countries.

## Supplementary Information

**Additional file 1: Table S1. **Associations between socio-demographic characteristics, historical sexual behaviour and post-traumatic stress symptoms (PC-PTSD-5 ≥3). **Table S2**. Associations between post-traumatic stress symptom score (PC-PTSD-5≥3 versus PC-PTSD-5<3) and sexual behaviour, PrEP attitudes and mental health co-morbidities.

## Data Availability

The analysis dataset will be made available upon request and accessed through the LSHTM Data Compass repository (https://datacompass.lshtm.ac.uk/).

## Abbreviations

CHAPS: Combined HIV Adolescent PrEP and Prevention Study
CI: Confidence interval
DSM-5: Diagnostic and Statistical Manual of Mental Disorders
GAD-2: Generalized Anxiety Disorder-2
MRC: Medical Research Council
OR: Odds ratio
PC-PTSD-5: Primary Care PTSD Screen for *DSM-5*
PHQ-9: Patient Health Questionnaire
PrEP: Pre-exposure prophylaxis
PTSD: Post traumatic stress disorder
SSA: Sub-Saharan Africa
