# Supplementary material for: Symptoms of post-traumatic stress and associations with sexual behaviour and PrEP preferences among young people in South Africa, Uganda and Zimbabwe
Source: BMC Infect Dis. 2022 May 16;22:466. doi: 10.1186/s12879-022-07430-2 (PMC9109411; doi:10.1186/s12879-022-07430-2)
Supplement: Supplementary file 1 — Additional file 1: Table S1. Associations between socio-demographic characteristics, historical sexual behaviour and post-traumatic stress symptoms (PC-PTSD-5 ≥3). Table S2. Associations between post-traumatic stress symptom score (PC-PTSD-5≥3 versus PC-PTSD-5<3) and sexual behaviour, PrEP attitudes and mental health co-morbidities. [file 12879_2022_7430_MOESM1_ESM.docx]

**Supplementary Table 1. Associations between socio-demographic characteristics, historical sexual behaviour and post-traumatic stress symptoms (PC-PTSD-5 ≥3)**

|  |  | **PC-PTSD score, categorised** | | **Crude results** | | **Adjusted results^1^** | |
| --- | --- | --- | --- | --- | --- | --- | --- |
| **Characteristic** | **Category** | **PC-PTSD-5 <3** | **PC-PTSD-5 ≥3** | **Odds ratio (95% CI)** | **p-value** | **Odds ratio (95% CI)** | **p-value** |
| Study setting | Cape Town | 174 (72.8%) | 65 (27.2%) | 1.95 (1.34, 2.83) | <0.001 | 2.04 (1.40, 2.98) | <0.001 |
|  | Johannesburg | 153 (76.5%) | 47 (23.5%) | 1.60 (1.07, 2.40) |  | 1.60 (1.06, 2.41) |  |
|  | Entebbe | 412 (83.9%) | 79 (16.1%) | Baseline |  | Baseline |  |
|  | Chitungwiza | 337 (84.3%) | 63 (15.8%) | 0.97 (0.68, 1.40) |  | 0.98 (0.68, 1.40) |  |
| Sex | Male | 570 (84.7%) | 103 (15.3%) | Baseline | <0.001 | Baseline | <0.001 |
|  | Female | 506 (77.0%) | 151 (23.0%) | 1.65 (1.25, 2.18) |  | 1.68 (1.27, 2.22) |  |
| Age group | 13-15 | 130 (86.7%) | 20 (13.3%) | 0.59 (0.36, 0.97) | 0.05 | 0.55 (0.33, 0.91) | 0.02 |
|  | 16-17 | 201 (83.8%) | 39 (16.3%) | 0.74 (0.51, 1.08) |  | 0.73 (0.50, 1.07) |  |
|  | 18-24 | 745 (79.3%) | 195 (20.7%) | Baseline |  | Baseline |  |
| Highest level of education attended | Still studying | 574 (82.5%) | 122 (17.5%) | Baseline | 0.10 | Baseline | 0.58 |
|  | ≤ Grade 7 | 109 (85.2%) | 19 (14.5%) | 0.82 (0.49, 1.39) |  | 0.89 (0.50, 1.59) |  |
|  | Grade 7-12 | 358 (78.0%) | 101 (22.0%) | 1.33 (0.99, 1.78) |  | 1.17 (0.84, 1.64) |  |
|  | Post-school | 35 (74.5%) | 12 (25.5%) | 1.61 (0.81, 3.20) |  | 1.42 (0.69, 2.89) |  |
| Participant is household head | No | 946 (81.1%) | 220 (18.9%) | Baseline | 0.57 | Baseline | 0.08 |
|  | Yes | 130 (79.3%) | 34 (20.7%) | 1.12 (0.75, 1.69) |  | 1.51 (0.96, 2.36) |  |
| Household head age | Per unit increase |  |  | 1.01 (1.00, 1.02) | 0.03 | 1.01 (1.00, 1.02) | 0.05 |
| Number of adults in household^2^ | 1-2 | 333 (83.9%) | 64 (16.1%) | Baseline | 0.08 | Baseline | 0.24 |
|  | 3-4 | 503 (80.9%) | 119 (19.1%) | 1.23 (0.88, 1.72) | 0.02^6^ | 1.20 (0.84, 1.73) | 0.09^6^ |
|  | 5+ | 239 (77.1%) | 71 (22.9%) | 1.55 (1.06, 2.52) |  | 1.42 (0.94, 2.13) |  |
| Number of rooms in household | 1-2 | 381 (83.7%) | 74 (16.3%) | Baseline | 0.13 | Baseline | 0.29 |
|  | 3-4 | 338 (80.3%) | 83 (19.7%) | 1.26 (0.89, 1.79) | 0.05^6^ | 1.23 (0.85, 1.77) | 0.13^6^ |
|  | 5+ | 357 (78.6%) | 97 (21.4%) | 1.40 (1.00, 1.96) |  | 1.34 (0.92, 1.94) |  |
| Number of adults per room in household^2^ | <1 | 435 (80.7%) | 104 (19.3%) | Baseline | 0.71 | Baseline | 0.68 |
|  | ≥1 and <2 | 426 (80.2%) | 105 (19.8%) | 1.03 (0.76, 1.39) |  | 1.05 (0.77, 1.44) |  |
|  | ≥2 | 214 (82.6%) | 45 (17.4%) | 0.88 (0.60, 1.29) |  | 0.88 (0.58, 1.38) |  |
| Ever had sex^3^ | No | 244 (87.1%) | 36 (12.9%) | Baseline | 0.003 | Baseline | 0.46 |
|  | Yes | 831 (79.4%) | 216 (20.6%) | 1.76 (1.20, 2.58) |  | 1.18 (0.75, 1.85) |  |
| Age of first sex | Per unit increase |  |  | 0.98 (0.93, 1.04) | 0.59 | 0.96 (0.90, 1.03) | 0.29 |
| Transactional sex, ever^4^ | No | 979 (81.9%) | 216 (18.1%) | Baseline | 0.01 | Baseline | 0.01 |
|  | Yes | 92 (72.4%) | 35 (27.6%) | 1.72 (1.14, 2.61) |  | 1.79 (1.16, 2.77) |  |
| Forced sex, last 6 months^5^ | No | 1031 (82.5%) | 219 (17.5%) | Baseline | <0.001 | Baseline | <0.001 |
|  | Yes | 43 (56.6%) | 33 (43.4%) | 3.61 (2.24, 5.82) |  | 3.62 (2.22, 5.90) |  |
| Forcing sex, last 6 months^5^ | No | 1044 (81.9%) | 231 (18.1%) | Baseline | <0.001 | Baseline | <0.001 |
|  | Yes | 30 (58.9%) | 21 (41.2%) | 3.16 (1.78, 5.63) |  | 3.48 (1.92, 6.32) |  |

^1^Adjusted for study setting, sex and age group;  ^2^1 missing value; ^3^3 preferred not to say; ^4^8 preferred not to say; ^5^4 preferred not to say; ^6^test for trend

**Supplementary Table 2. Associations between post-traumatic stress symptom score (PC-PTSD-5 ≥3 versus PC-PTSD-5<3) and sexual behaviour, PrEP attitudes and mental health co-morbidities**

|  |  | | | | | | | | | |  | | | | | | | **Crude results** | | **Adjusted for study setting, sex and age group** | | **Further adjusted for depression and anxiety** | |
| --- | --- | --- | --- | --- | --- | --- | --- | --- | --- | --- | --- | --- | --- | --- | --- | --- | --- | --- | --- | --- | --- | --- | --- |
|  |  | | | | | | | | | |  | | | | | | | **OR (95% CI)** | **p-value** | **OR (95% CI)** | **p-value** | **OR (95% CI)** | **p-value** |
| **SEXUAL BEHAVIOUR CHARACTERISTICS** | | | | | | | | | | | | | | | | | |  |  |  |  |  |  |
| ***Outcome: Number partners, last 6 months*** | | | | | | | | | | | | | | | | | |  |  |  |  |  |  |
| **PTSD group** | **0** | | | **1** | | | | | | | **2** | | | | | **3+** | |  |  |  |  |  |  |
| PC-PTSD-5<3 | 313 (29.3%) | | | 413 (38.7%) | | | | | | | 168 (15.8%) | | | | | 173 (16.2%) | | Baseline | 0.003 | Baseline | 0.14 | Baseline | 0.19 |
| PC-PTSD-5≥3 | 51 (20.3%) | | | 103 (41.0%) | | | | | | | 42 (16.7%) | | | | | 55 (21.9%) | | 1.46 (1.14, 1.87) |  | 1.22 (0.94, 1.59) |  | 1.19 (0.91, 1.56) |  |
| ***Outcome: Sex frequency, past month*** | | | | | | | | | | | | | | | | | |  |  |  |  |  |  |
| **PTSD group** | **Never** | | | | | | **Weekly-monthly** | | | | | | | **>Weekly** | | | |  |  |  |  |  |  |
| PC-PTSD-5<3 | 464 (44.7%) | | | | | | 359 (34.6%) | | | | | | | 214 (20.6%) | | | | Baseline | 0.001 | Baseline | 0.28 | Baseline | 0.36 |
| PC-PTSD-5≥3 | 83 (35.0%) | | | | | | 82 (34.6%) | | | | | | | 72 (30.4%) | | | | 1.58 (1.21, 2.05) |  | 1.17 (0.88, 1.55) |  | 1.14 (0.86, 1.52) |  |
| ***Outcome: Last time had sex, how far in advance knew*** | | | | | | | | | | | | | | | | | |  |  |  |  |  |  |
| **PTSD group** | **>24 hours** | | **13-24 hours** | | | | | | **2-12 hours** | | | | | | **<2 hours** | | |  |  |  |  |  |  |
| PC-PTSD-5<3 | 226 (27.2%) | | 74 (8.9%) | | | | | | 155 (18.7%) | | | | | | 376 (45.3%) | | | Baseline | 0.07 | Baseline | 0.18 | Baseline | 0.18 |
| PC-PTSD-5≥3 | 41 (19.0%) | | 17 (7.9%) | | | | | | 53 (24.5%) | | | | | | 105 (48.6%) | | | 1.29 (0.98, 1.70) |  | 1.21 (0.91, 1.60) |  | 1.22 (0.91, 1.62) |  |
| ***Outcome: Condom use last sex with recent partner*** | | | | | | | | | | | | | | | | | |  |  |  |  |  |  |
| **PTSD group** | **No** | | | | | | | | **Yes** | | | | | | | | |  |  |  |  |  |  |
| PC-PTSD-5<3 | 417 (50.2%) | | | | | | | | 414 (49.8%) | | | | | | | | | Baseline | 0.11 | Baseline | 0.13 | Baseline | 0.31 |
| PC-PTSD-5≥3 | 121 (56.3%) | | | | | | | | 94 (43.7%) | | | | | | | | | 0.78 (0.58, 1.06) |  | 0.78 (0.57, 1.08) |  | 0.85 (0.62, 1.17) |  |
| ***Outcome: Type of relationship, most recent partner*** | | | | | | | | | | | | | | | | | |  |  |  |  |  |  |
| **PTSD group** | **Regular** | | | | | | | | **Casual, paying or other** | | | | | | | | |  |  |  |  |  |  |
| PC-PTSD-5<3 | 688 (82.8%) | | | | | | | | 143 (17.2%) | | | | | | | | | Baseline | 0.54 | Baseline | 0.17 | Baseline | 0.23 |
| PC-PTSD-5≥3 | 175 (81.0%) | | | | | | | | 41 (19.0%) | | | | | | | | | 1.13 (0.77, 1.66) |  | 1.33 (0.89, 2.00) |  | 1.29 (0.86, 1.95) |  |
| ***Outcome: HIV status, most recent partner*** | | | | | | | | | | | | | | | | | |  |  |  |  |  |  |
| **PTSD group** | **Negative** | | | | | | | | **Positive or don’t know** | | | | | | | | |  |  |  |  |  |  |
| PC-PTSD-5<3 | 483 (58.3%) | | | | | | | | 346 (41.7%) | | | | | | | | | Baseline | 0.52 | Baseline | 0.16 | Baseline | 0.16 |
| PC-PTSD-5≥3 | 120 (55.8%) | | | | | | | | 95 (44.2%) | | | | | | | | | 1.11 (0.82, 1.50) |  | 1.26 (0.92, 1.72) |  | 1.26 (0.92, 1.73) |  |
| **GENERAL RISK TAKING AND HIV SALIENCE CHARACTERISTICS** | | | | | | | | | | | | | | | | | |  |  |  |  |  |  |
| ***Outcome: Self-perception of frequency of risk-taking (3 category Likert scale)*** | | | | | | | | | | | | | | | | | | |  |  |  |  |  |
| **PTSD group** | **Never** | | | | | **Sometimes** | | | | | | | **Often** | | | | |  |  |  |  |  |  |
| PC-PTSD-5<3 | 638 (59.3%) | | | | | 239 (22.2%) | | | | | | | 199 (18.5%) | | | | | Baseline | <0.001 | Baseline | 0.001 | Baseline | 0.003 |
| PC-PTSD-5≥3 | 112 (44.1%) | | | | | 79 (31.1%) | | | | | | | 63 (24.8%) | | | | | 1.70 (1.32, 2.20) |  | 1.60 (1.22, 2.10) |  | 1.53 (1.16, 2.01) |  |
| ***Outcome: How often thought about risk of HIV, last 3 months*** | | | | | | | | | | | | | | | | | | |  |  |  |  |  |
| **PTSD group** | **Never** | | **Rarely** | | | | | | **Sometimes** | | | | | | **Often** | | |  |  |  |  |  |  |
| PC-PTSD-5<3 | 633 (58.8%) | | 153 (14.2%) | | | | | | 247 (23.0%) | | | | | | 43 (4.0%) | | | Baseline | <0.001 | Baseline | <0.001 | Baseline | <0.001 |
| PC-PTSD-5≥3 | 105 (41.3%) | | 40 (15.8%) | | | | | | 88 (34.7%) | | | | | | 21 (8.3%) | | | 2.04 (1.58, 2.64) |  | 1.85 (1.41, 2.42) |  | 1.70 (1.29, 2.24) |  |
| ***Outcome: Chance of HIV, next 3 months*** | | | | | | | | | | | | | | | | | |  |  |  |  |  |  |
| **PTSD group** | **None** | | | | | **Some** | | | | | | | **Moderate-high** | | | | |  |  |  |  |  |  |
| PC-PTSD-5<3 | 706 (65.6%) | | | | | 289 (26.9%) | | | | | | | 81 (7.5%) | | | | | Baseline | 0.003 | Baseline | 0.09 | Baseline | 0.18 |
| PC-PTSD-5≥3 | 144 (56.7%) | | | | | 77 (30.3%) | | | | | | | 33 (13.0%) | | | | | 1.51 (1.15, 1.98) |  | 1.28 (0.97, 1.70) |  | 1.22 (0.92, 1.62) |  |
| **ATTITUDES TOWARDS PrEP** | | | | | | | | | | | | | | | | | |  |  |  |  |  |  |
| ***Outcome: PrEP preference*** | | | | | | | | | | | | | | | | | |  |  |  |  |  |  |
| **PTSD group** | | **On demand** | | | | | | **Daily** | | | | | | | | | |  |  |  |  |  |  |
| PC-PTSD-5<3 | | 639 (61.4%) | | | | | | 402 (38.6%) | | | | | | | | | | Baseline | 0.005 | Baseline | 0.10 | Baseline | 0.11 |
| PC-PTSD-5≥3 | | 127 (51.6%) | | | | | | 119 (48.4%) | | | | | | | | | | 1.49 (1.13, 1.97) |  | 1.29 (0.95, 1.74) |  | 1.28 (0.94, 1.73) |  |
| ***Outcome: Would take PrEP if same price as hot meal*** | | | | | | | | | | | | | | | | | |  |  |  |  |  |  |
| **PTSD group** | **No** | | | | | | | | | **Yes** | | | | | | | |  |  |  |  |  |  |
| PC-PTSD-5<3 | 324 (30.1%) | | | | | | | | | 752 (69.9%) | | | | | | | | Baseline | 0.42 | Baseline | 0.72 | Baseline | 0.50 |
| PC-PTSD-5≥3 | 70 (27.6%) | | | | | | | | | 184 (72.4%) | | | | | | | | 1.13 (0.84, 1.54) |  | 1.06 (0.77, 1.45) |  | 1.12 (0.81, 1.54) |  |
| ***Outcome: Would disclose PrEP to partner*** | | | | | | | | | | | | | | | | | |  |  |  |  |  |  |
| **PTSD group** | **No** | | | | | | | | **Yes** | | | | | | | | |  |  |  |  |  |  |
| PC-PTSD-5<3 | 350 (33.8%) | | | | | | | | 786 (66.3%) | | | | | | | | | Baseline | 0.90 | Baseline | 0.61 | Baseline | 0.88 |
| PC-PTSD-5≥3 | 80 (33.3%) | | | | | | | | 160 (66.7%) | | | | | | | | | 1.02 (0.76, 1.37) |  | 0.92 (0.68, 1.26) |  | 0.98 (0.71, 1.33) |  |
| **ALCOHOL AND DRUG USE** | | | | | | | | | | | | | | | | | |  |  |  |  |  |  |
| ***Outcome: alcohol use, frequency of binge drinking*** | | | | | | | | | | | | | | | | | |  |  |  |  |  |  |
| **PTSD group** | **Don’t drink** | | | | **Drink, no binge** | | | | | | | **< Monthly** | | | | | **≥ Monthly** |  |  |  |  |  |  |
| PC-PTSD-5<3 | 595 (55.5%) | | | | 152 (14.2%) | | | | | | | 139 (13.0%) | | | | | 187 (17.4%) | Baseline | <0.001 | Baseline | 0.03 | Baseline | 0.04 |
| PC-PTSD-5≥3 | 108 (42.7%) | | | | 37 (14.6%) | | | | | | | 41 (16.2%) | | | | | 67 (26.5%) | 1.69 (1.31, 2.18) |  | 1.37 (1.04, 1.81) |  | 1.35 (1.02, 1.78) |  |
| ***Outcome: Drug use, last 30 days*** | | | | | | | | | | | | | | | | | |  |  |  |  |  |  |
| **PTSD group** | **No** | | | | | | | | **Yes** | | | | | | | | |  |  |  |  |  |  |
| PC-PTSD-5<3 | 950 (88.3%) | | | | | | | | 126 (11.7%) | | | | | | | | | Baseline | 0.04 | Baseline | 0.18 | Baseline | 0.31 |
| PC-PTSD-5≥3 | 212 (83.5%) | | | | | | | | 42 (16.5%) | | | | | | | | | 1.49 (1.02, 2.18) |  | 1.34 (0.88, 2.06) |  | 1.25 (0.81, 1.94) |  |
